# Further refinement of surgery will not necessarily improve outcome after hip fracture

**DOI:** 10.1080/17453674.2019.1706936

**Published:** 2020-01-08

**Authors:** Cecilia Rogmark

**Affiliations:** Department of Orthopaedics, Skåne Univerisity Hospital, Malmö, Sweden

A displaced femoral neck fracture needs urgent surgery. More than 10 years ago hip replacement was found to be beneficial over internal fixation (Rogmark and Johnell 2007), but we still debate whether it should be a hemiarthroplasty (HA) or a total hip arthroplasty (THA).

In a recent issue of *Acta Orthopaedica*, Hansson et al. (2019) pinpoint the dilemma: THA as fracture treatment is associated with more hip complications, mainly dislocations. But our research group has previously noted fewer revision surgeries after THA compared with HA (Hansson et al. 2017). So did 2 other register studies, from Canada and UK (Ravi et al. 2019; Metcalfe et al. 2019). In contrast, Dutch register data found THA to be associated with a higher revision rate (Moerman et al. 2018). Yet another UK register paper had similar revision rates for the 2 methods (Jameson et al. 2013), but they supported the current Swedish study (Hansson et al. 2019) when describing a higher dislocation rate after THA.

Can the clinical studies guide us? The HEALTH study is an ambitious international project (HEALTH Investigators 2019). This randomized trial, including 1,495 patients, cannot show any clear differences between the 2 methods. The somewhat better functional results after THA may be outweighed by slightly more complications. However, one has to question the external validity of the HEALTH study. On average, just over 2 patients per each of the 80 participating hospitals and years were recruited during the study period. So few, from an otherwise large patient group, signals a selection bias. A downside of an HA may be development of acetabular erosion. Both the short-term follow-up of 2 years and the lack of radiological follow-up mean that this condition is not covered at all by the HEALTH study. Other randomized trials on the topic confirm a lower reoperation risk but higher dislocation risk after THA, without any differences in mortality or infection. In terms of patient-reported outcome, THA leads to better results (in terms of functional scores) than HA in some, but not all, RCTs (Lewis et al. 2019).

Three things may explain the contradictory results: selection bias, performance bias, or simply that the implant does not matter that much.

Many of the register papers find an association between THA and lower mortality. It is not plausible that an added acetabular cup per se should protect against death. It would take a substantial gain in functional outcome after THA, compared with HA, to affect general health and risk of dying. If anything, the longer surgery and higher blood loss associated with THA would increase the risk of death. Even after adjusting for comorbidity and other factors, residual confounding seems to explain the lower mortality after THA, as discussed by Hansson et al. (2019). Choice of implant is most likely influenced by the patient’s degree of frailty and physical activity, factors not available in any register. Frail and incapacitated individuals, more often selected for HA, also suffer a higher risk of dying. An indication that the surgical procedure in itself is not decisive for the risk of dying is that mortality rates after femoral neck fracture have been unchanged for the last 3 decades (Mundi et al. 2014)—a period that has seen many surgical developments taking place.

A worse general condition of the typical HA patient will also interfere with the choice to perform revision surgery in case of any complication. The frailer the patient, the more reluctant she and/or the surgeon will be to revise the implant. On the other hand, we may have a lower threshold to revise an HA than a THA. In the case of dislocation or erosion, conversion of HA to THA appears relatively easy, by adding a socket. A troublesome THA, without apparent malpositioning, might more often be left in place. In any case, revision is a very blunt outcome measure after arthroplasty in the fracture population. Dislocation—more common after THA in both register and clinical studies—is a serious complication for the elderly. A second dislocation leads to a permanent loss of health-related quality-of-life (Enocson et al. 2009). The increased risk of hip complications in general, and dislocation in particular should make us think twice before widening the indications for THA as fracture treatment.

Arthroplasty surgeons are more often proponents of THA. In fracture cases, the THA procedure is technically demanding on the surgeon and a high annual volume is needed for a good result. HA is considered to be more “forgiving” surgery. As hip fracture surgery may be done outside office hours and is often considered a newcomer’s task, an easy surgical technique is preferable. Hospitals may have an acute or elective profile, which may influence the result of scientific studies.

I strongly believe that now is the time for us as orthopedic surgeons to raise our heads from the detailed comparisons of 2 well-functioning surgical procedures and take more responsibility for the entire clinical pathway. Together with the multidisciplinary team of nurses, geriatricians, physiotherapists, GPs, and others, we have to work in an evidence-based manner to support the individual’s recovery from a hip fracture (Kammerlander et al. 2010). The aforementioned unchanged mortality is one indication that things outside surgery should be done better! Any subtle gain in function and satisfaction due to THA surgery will be lost if post-discharge rehabilitation is not structured and provided over several months.

After leaving the operating theater, there is still a lot left to do!
